# Local Directional Probability Optimization for Quantification of Blurred Gray/White Matter Junction in Magnetic Resonance Image

**DOI:** 10.3389/fncom.2017.00083

**Published:** 2017-09-12

**Authors:** Xiaoxia Qu, Jian Yang, Danni Ai, Hong Song, Luosha Zhang, Yongtian Wang, Tingzhu Bai, Wilfried Philips

**Affiliations:** ^1^Beijing Engineering Research Center of Mixed Reality and Advanced Display, School of Optics and Electronics, Beijing Institute of Technology Beijing, China; ^2^Department of Telecommunications and Information Processing (imec-IPI-TELIN), Ghent University Ghent, Belgium; ^3^School of Software, Beijing Institute of Technology Beijing, China; ^4^Academy of Opto-Electronics, Chinese Academy of Sciences Beijing, China

**Keywords:** epilepsy, focal cortical dysplasia, magnetic resonance images, blurred gray/white matter junction, feature computation

## Abstract

The blurred gray/white matter junction is an important feature of focal cortical dysplasia (FCD) lesions. FCD is the main cause of epilepsy and can be detected through magnetic resonance (MR) imaging. Several earlier studies have focused on computing the gradient magnitude of the MR image and used the resulting map to model the blurred gray/white matter junction. However, gradient magnitude cannot quantify the blurred gray/white matter junction. Therefore, we proposed a novel algorithm called local directional probability optimization (LDPO) for detecting and quantifying the width of the gray/white matter boundary (GWB) within the lesional areas. The proposed LDPO method mainly consists of the following three stages: (1) introduction of a hidden Markov random field-expectation-maximization algorithm to compute the probability images of brain tissues in order to obtain the GWB region; (2) generation of local directions from gray matter (GM) to white matter (WM) passing through the GWB, considering the GWB to be an electric potential field; (3) determination of the optimal local directions for any given voxel of GWB, based on iterative searching of the neighborhood. This was then used to measure the width of the GWB. The proposed LDPO method was tested on real MR images of patients with FCD lesions. The results indicated that the LDPO method could quantify the GWB width. On the GWB width map, the width of the blurred GWB in the lesional region was observed to be greater than that in the non-lesional regions. The proposed GWB width map produced higher F-scores in terms of detecting the blurred GWB within the FCD lesional region as compared to that of FCD feature maps, indicating better trade-off between precision and recall.

## Introduction

The blurred gray/white matter junction in T1-weighted magnetic resonance (MR) image is an important characteristic of focal cortical dysplasia (FCD) (Bernasconi et al., 2001; Antel et al., 2002, 2003; Bernasconi and Bernasconi, 2011). FCD is a localized malformation of brain cortical development and is the main cause of epilepsy in 30% of the patients (Bernasconi and Bernasconi, 2011). The World Health Organization has reported approximately 50 million people to be suffering from epilepsy globally (Bernasconi and Bernasconi, 2011). FCD lesions can be surgically treated. Notably, the lesions must be located prior to the surgery. MR imaging is a non-invasive imaging technique and is the most important tool in the pre-surgical evaluation of FCD lesions. The detection of FCD lesions on MR image depends on the feature analysis of the lesions; thus, computing and analyzing features of the lesions using MR images are important for diagnosing and treating FCD lesions.

Several features have been proposed to enhance the differences between lesional and non-lesional regions. The main features of the lesions on T1-weighted MR image are increased cortical thickness, blurred gray/white matter junction, and hyper-intensity (Bernasconi et al., 2001). A gray matter (GM) thickness map (Antel et al., 2002) and z-score of GM thickness map (Colliot et al., 2006) have been proposed for the analysis of increased cortical thickness. Gradient map is applied to model the blurred gray/white matter junction (Bernasconi et al., 2001), and the relative intensity map (Bernasconi et al., 2001) is presented to detect hyper-intensity. Ratio (Bernasconi et al., 2001) and composite (Antel et al., 2002) maps have been proposed to analyze synthetically the increased cortical thickness, blurred gray/white matter junction, and hyper-intensity. Complex map (Rajan et al., 2009), sulcal depth (Besson et al., 2008a,b,c), curvature (Besson et al., 2008a,b,c), fuzzy C-means index matrix (Shen et al., 2011), fractional anisotropy (Strumia et al., 2013), and skewness (Strumia et al., 2013) have also been proposed to increase the contrast between lesional and non-lesional regions. Features that are widely applied to pattern recognition in the image-processing field have great potential in FCD lesion detection, considering that lesion detection follows the same principle as that of pattern recognition. Therefore, image features or textures have also been investigated to detect FCD lesions, including texture features based on gray-level co-occurrence matrix (Haralick et al., 1973; Antel et al., 2003; Loyek et al., 2008), statistic feature (Loyek et al., 2008), and gray run-length matrix feature (Loyek et al., 2008).

Several earlier studies have focused on FCD lesional features, but only a few have focused on the features of the blurred gray/white matter junction (Bernasconi et al., 2001; Antel et al., 2002; Huppertz et al., 2005; Qu et al., 2014). Bernasconi et al. (2001) proposed to compute the absolute intensity gradient of the gray image for analyzing the blurred gray/white matter junction. The gradient values are obtained from the first-order derivative within a 5 × 5 × 5 local window. The resultant values demonstrate a steep gradient for the normal gray/white matter junction and a gradual one for the blurred gray/white matter junction. Therefore, small gradient values on the gradient map correspond to the blurred gray/white matter junction. Antel et al. (2002) proposed to execute a convolution of three-dimensional (3D) Gaussian function on MR image and use the resulting gradient values to form the gradient map. Huppertz et al. (2005) proposed a junction image to enhance the visibility of the blurred gray/white matter junction. The input image is first normalized to obtain the junction image. The region between GM and white matter (WM), which is called gray/white matter junction, is then segmented in accordance with the thresholds related to the mean and standard deviation values of GM and WM. The binary image, in which the gray/white matter junction is denoted by 1 and other regions by 0, is processed as a 3D convolution resulting in a convolved image. The junction image is constructed through the comparison between the convolved image of an individual and the mean convolved images in the normal database. In the junction image, the blurred gray/white matter junction is brighter than the normal gray/white matter junction.

In general, the existing methods for analyzing the blurred gray/white matter junction have successfully enhanced the difference between lesional and non-lesional regions. However, the gradient values generated using the existing methods depend on the image gray values and cannot be utilized to quantify the lesions. Thus, the gradient values cannot reflect the number of physical spaces occupied by the blurred gray/white matter junction and the expected width of the normal gray/white matter junction.

**Figure 1:**
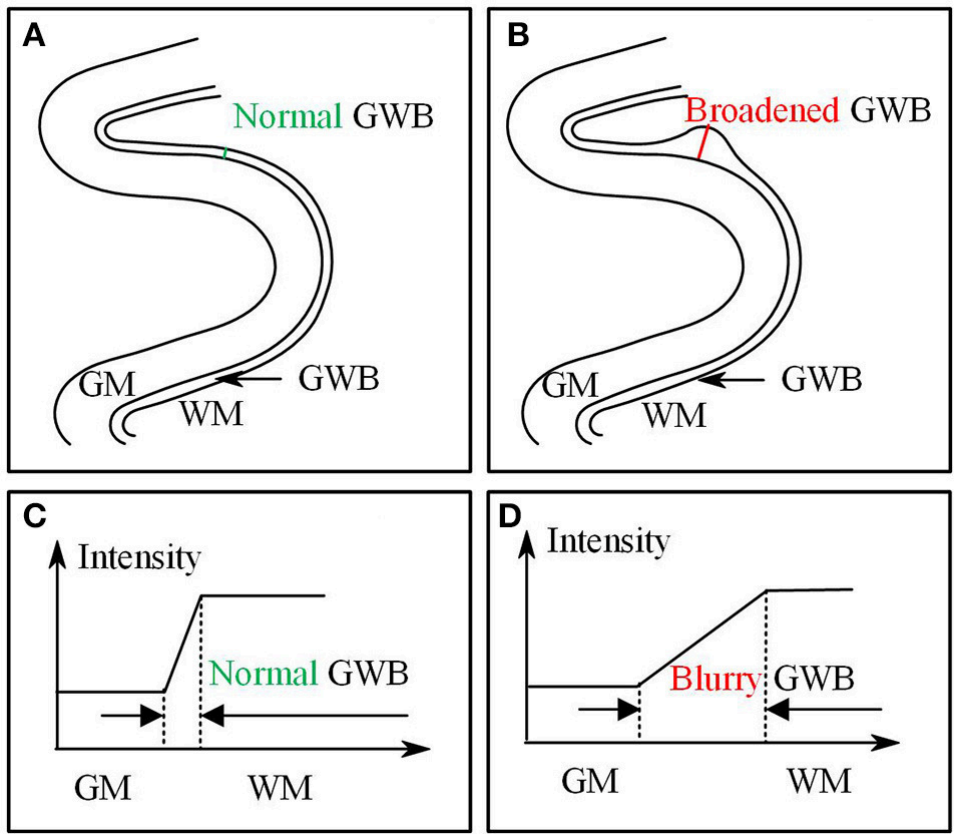
Images present the normal gray/white matter boundary (GWB) **(A,C)** and focal cortical dysplasia (FCD) region **(B,D)** with blurred GWB. The intensities on blurry region vary slowly as shown in **(C)**, while they vary sharp on normal GWB region as shown in **(D)**. When look at the GWB in space, the blurred GWB is wider than the normal GWB (**A** vs. **B**), indicating that the blurred GWB can be also considered as broadened GWB.

Based on the results of previous studies measuring cortical thickness (Jones et al., 2000; Hutton et al., 2008), we proposed to measure quantitatively the blurring of the gray/white matter boundary (GWB). Although the present study is similar to a previous work (Jones et al., 2000), the cortical thickness study focused on the GM region, while the present GWB-width study focused on the junction between the GM and WM. The GM and GWB regions appear different because their intensity changes (Figures 1C,D) and locations (Figures 1A,B) are different. We considered the blurred gray/white matter junction as a broadened GWB to quantify its features, as shown in Figure 1. The brain MR image is mainly composed of GM, WM, and cerebral spinal fluid (CSF); the GWB is located between the GM and WM. Regarding the gray level variation, the gray values in the normal GWB increase from the GM to the WM value, as shown in Figure 1C. The value gradient that increases from the GM to the WM is smaller in the blurry GWB region than that in the non-blurred GWB region, as shown in Figure 1D. With respect to the location variation, the blurred GWB region is wider than the normal GWB region, as shown in Figures 1A,B. Hence, the blurred GWB can also be considered as a broadened GWB region.

In the present study, we proposed local directional probability optimization (LDPO) as a new method to quantify the gray/white matter junction by measuring the GWB-width. Three concerns arose with respect to the computation of the GWB width: (1) obtaining the GWB region from the brain MR images, (2) generating local directions from GM to WM in the GWB region passing voxels on GWB, and (3) finding the optimal local directions for estimating the GWB width values. Our key contributions were three-fold. We addressed the first concern by introducing the hidden Markov random field-expectation-maximization (HMRF-EM) algorithm (Zhang et al., 2001) in our analysis. The second problem was solved through considering the GWB region as an electric potential field, based on the work of Jones et al. (2000). The solution to the third problem was based on iterative neighborhood search and finding the optimal local path, which could be used to compute the GWB width.

We presented preliminary versions of the proposed methods at EMBS 2014 (Qu et al., 2014). In the conference paper, we mainly described the determination of the optimal directions and measurement of the GWB width based on the path of optimal directions. The comparison results of GWB width and gradient maps were also presented. In the present study, we have added more details on the measurement of the GWB region and the acquisition of the local directions. In addition to comparison with the gradient map, the proposed GWB width map was also compared to other FCD lesional features. The image and evaluation results were also described in more contents in this study. The application of the GWB width map obtained from the proposed LDPO method together with other FCD features to automated detection of FCD lesions has been described in another study (Qu et al., 2016).

## Methods

### Datasets and ground truth

We studied the T1-weighted MR images of 10 patients (P1–P10) with FCD lesions (one scan per patient) (Qu et al., 2014, 2016). The scans were acquired at the Ghent University Hospital on a Siemens 3T MR scanner. Each scan consisted of 256 × 256 × 176 voxel matrices with a resolution of 0.8594 × 0.8594 × 0.9 mm^3^.

All patients were diagnosed by a neuroradiologist (Dr. Karel Deblaere from Ghent University Hospital) and his colleagues. Patients were diagnosed at the clinic using T1-weighted and fluid-attenuated inversion recovery (FLAIR) images after correlation with clinical symptoms; lesions were histopathologically confirmed after surgery. FCD lesions were delineated by the neuroradiologist using active contour segmentation of the ITK-SNAP^1^ tool (Yushkevich et al., 2006) on the axial views of the T1-weighted with the auxiliary information of FLAIR MR images. The segmentations were manually adjusted as necessary. The delineations on the T1-weighted images were used as the ground truth to evaluate the proposed GWB width map as a lesional feature.

### Preprocessing

Preprocessing aims to refine the original raw image and prepare the images for further analysis. The brain extraction tool proposed by Smith (2002) was applied to extract the brain region on the T1-weighted MR images because the FCD lesions occur only in the brain. The intensity of non-uniformity (also called bias field) was corrected using a modified (EM) algorithm (Zhang et al., 2001) to obtain consistent intensities of the extracted brain region. The intensity ranges of all images were then normalized between 0 and 255 using the method proposed by Nyul and Udupa (1999).

We first used rigid registration to roughly align images to unify their spaces. The affine registration was then used to further standardize the images. The reference image for registration was the MNI152 brain T1-weighted MR image^2^ from the Montreal Neurological Institute. We could only align the general brain regions, without matching details such as gyral GM regions, as the brains of different patients have different topological structures. We scaled the brain regions into the same size using this method, while preserving the topological structure and details. As a result, the differences in total brain volume among different patients were adjusted.

In the registered images, the brain tissues that were not anatomically related to FCD lesions (brain cerebellum, brain stem, striatum, and thalamus) were eliminated. The brain atlas of images from the Montreal Neurological Institute (MNI) (Mazziotta et al., 2001; Diedrichsen et al., 2009) was taken as the template for elimination.

### LDPO for GWB width estimation

The proposed GWB width feature has been defined in this section. The three key concerns for computing the feature, namely, (1) obtaining or segmenting the GWB from the brain MR images (Section Probabilities of Brain Tissues for GWB Segmentation), (2) generating local directions from GM to WM passing through voxels in the GWB region (Section Local Direction for the Generation of Paths from GM to WM), and (3) finding the optimal local directions for estimating the GWB width values (Section Local Directional Optimization for GWB Width Estimation), have been described. Moreover, the solutions to the concerns have also been described.

#### Definition of the GWB width

**Figure 2:**
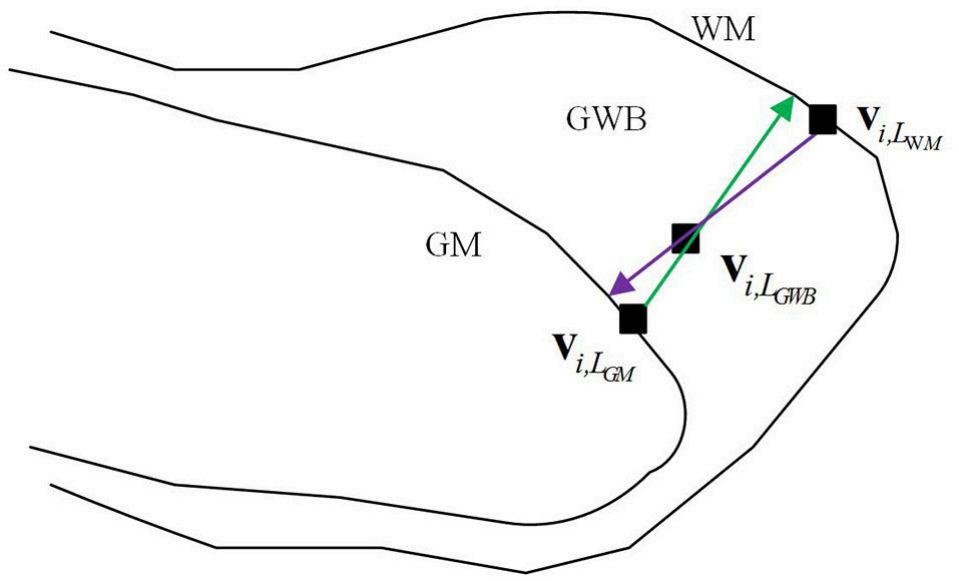
Definition of the width at voxels within GWB region.

The definition of the GWB width has been illustrated in Figure 2. The GWB width aims to describe the shortest distance between the GM and WM as the GWB region is located between these two regions. For each voxel within the GWB denoted by **v**_*i*,__*L*_*GWB*__, we found the voxel closest to the given voxel **v**_*i*,__*L*_*GWB*__, on the GM, denoted by **v**_*i*,__*L*_*GM*__. The **v**_*i*,__*L*_*GWB*__ and **v**_*i*,__*L*_*GM*__ form a direction from the GM to WM which is indicated by a green line with an arrow in Figure 2. Similarly, we can also find the closest voxel to the given voxel **v**_*i*,__*L*_*GWB*__ on the WM denoted by **v**_*i*,__*L*_W*M*__. The **v**_*i*,__*L*_*GWB*__ and **v**_*i*,__*L*_W*M*__ also form a direction from WM to GM which is denoted by a purple line with an arrow in Figure 2. Considering that the direction from **v**_*i*,__*L*_*GWB*__ to **v**_*i*,__*L*_*GM*__ and the direction from **v**_*i*,__*L*_*GWB*__ to **v**_*i*,__*L*_W*M*__ may not be collinear, we computed the lengths of the green line and purple line in Figure 2, and calculated their mean value as the final GWB width at the given voxel **v**_*i*,__*L*_*GWB*__.

The computation of the GWB width at **v**_*i*,__*L*_*GWB*__ denoted by *D*(**v**_*i*,__*L*_*GWB*__) is as follows:

(1) $$\begin{array}{l} D\left(v_{i,L_{GWB}}\right)=(d\left(v_{i,L_{GM}},v_{i,L_{WM}}\right){|}_{v_{i,L_{GM}}\to v_{i,L_{GWB}}}\\ \;+d\left(v_{i,L_{WM}},v_{i,L_{GM}}\right){|}_{v_{i,L_{GWB}}\to v_{i,L_{WM}}})/2 \end{array}$$

where $d\left(v_{i,L_{GM}},v_{i,L_{WM}}\right){|}_{v_{i,L_{GM}}\to v_{i,L_{GWB}}}$ is the Euclidean distance between **v**_*i*,__*L*_*GM*__ and **v**_*i*,__*L*_*WM*__ along the direction from **v**_*i*,__*L*_*GM*__ to **v**_*i*,__*L*_*GWB*__ (green line with arrow in Figure 2); $d\left(v_{i,L_{WM}},v_{i,L_{GM}}\right){|}_{v_{i,L_{GWB}}\to v_{i,L_{WM}}}$ is the distance between **v**_*i*,__*L*_W*M*__ and **v**_*i*,__*L*_*GM*__ along the direction from **v**_*i*,__*L*_*GWB*__ to **v**_*i*,__*L*_W*M*__ (purple line with arrow in Figure 2).

The value of GWB width at **v**_*i*,__*L*_*GWB*__ describes the distance from GM to WM, and the value could be used to estimate whether blurry GM/WM matter junction (broaden GWB) existed.

#### Probabilities of brain tissues for GWB segmentation

This subsection presents how the GWB region was obtained on the brain MR images. The GWB region was defined as the region belonging partly to the GM and partly to the WM based on the intensities and locations. The spatial information of the brain tissues was essential to achieve the goal. The HMRF-EM algorithm (Zhang et al., 2001) was introduced to analyze the spatial information of brain tissues and to generate the probabilities of brain tissues, including the GM, WM, and CSF. The labeled image containing the GM, WM, and GWB was then acquired based on the probabilities of brain tissues.

The probability of one tissue at one voxel indicated that multiple brain tissues may occupy one voxel, and the histograms of the probability of these tissues had overlapping parts. This phenomenon was also considered as a partial volume effect (Zhang et al., 2001). A probability image could be obtained from analyzing the gray-scale MR scan. The size of the probability image was equal to the input MR image. In the probability image of one brain tissue, the value of each voxel represented the proportion of the brain tissue in that voxel. For example, if one voxel on the probability image of GM had a value of 0.7, then 70% of the voxel belonged to the GM and the remaining 30% to other brain tissues

Several symbols and terms need to be defined to compute the probability images. The input gray image is denoted by the vector *Y* = (*y* (**v**_1_), *y* (**v**_2_), …, *y* (**v**_*i*_), …, *y* (**v**_*N*_)), where **v**_*i*_ is the *i*-th voxel, *y* (**v**_*i*_) is the intensity value of the *i*-th voxel **v**_*i*_, and *N* is the total number of the voxels in the image. After the input image *Y* is processed, three probability images of GM, WM, and CSF are obtained and denoted by *P*_GM_, *P*_WM_, and *P*_CSF_, respectively. We obtain *P*_GM_ = (*p*_GM_ (**v**_1_), …, *p*_GM_ (**v**_*i*_), …, *p*_GM_ (**v**_*N*_)), *P*_WM_ = (*p*_WM_ (**v**_1_), …, *p*_WM_ (**v**_*i*_), …., *p*_WM_ (**v**_*N*_)), and *P*_CSF_ = (*p*_CSF_ (**v**_1_), …, *p*_*CSF*_ (**v**_*i*_), …, *p*_CSF_ (**v**_*N*_)). *p*_GM_ (**v**_*i*_) is the probability value at the *i*-th voxel **v**_*i*_ on the probability image of GM. *p*_WM_ (**v**_*i*_) and *p*_*CSF*_ (**v**_*i*_) have similar meaning as *p*_GM_ (**v**_*i*_).

The *P*_GM_, *P*_WM_, and *P*_CSF_ are generated as follows: image *Y* is first segmented into an image with labels denoted by *X* = (*x*(**v**_1_), *x*(**v**_2_), …, *x*(**v**_*i*_), …, *x*(**v**_*N*_)), where *X* is the segmented image of *Y*; *x*(**v**_*i*_) is the label value at the voxel **v**_*i*_, *x*(**v**_*i*_) ∈ **L**; **L** is the set of all labels. For example, if an image is segmented into a binary image, then **L** = {0, 1}. In this study, we obtain the **L** = {*L*_GM_, *L*_WM_, *L*_CSF_}, where *L*_GM_, *L*_WM_, and *L*_CSF_ are the labels of the GM, WM, and CSF, respectively.

The purpose of image segmentation is to find an image $\hat{\text{X}}$ that is the estimation of the image with true labels denoted by X^*^. The maximum a posteriori probability (Duda et al., 2000) indicates that the estimated labels should satisfy the following equation:

(2) $$\begin{array}{l} \hat{\text{X}}=\underset{\text{x}}{\text{arg max}}\left\{p\left(\text{Y}|\text{X},\Theta \right)p\left(\text{X}\right)\right\} \end{array}$$

where *p*(**X**) is the prior probability, *p*(**Y**|**X**, Θ) is the joint probability, and Θ is the set of parameters.

Markov random field (MRF) (Kindermann, 1980) (denoted by χ) is usually utilized to describe the neighboring information of voxels to make use of the spatial information for computing the prior probability *p*(**X**) (Geman and Geman, 1984; Li, 1995; Zhang et al., 2001). According to the MRF, spatial information of image can be encoded through cortex constraints of neighboring voxels. In this way, X can be taken as a realization of the MRF. Therefore, the prior probability is *p*(**X**) = (1/*Z*)exp(−*U*(*X*)), where *Z* is a normalized coefficient called partition function; *U*(*X*) is an energy function defined as $U\left(X\right)=\sum_{c\in C}V_{c}(X)$, where *c* is a clique which is composed of a subset of location set (definition of location set will be detailed described in the following paragraphs), *C* is the set of all possible cliques, *V*_*c*_(*X*) is the clique potential, and *U*(*X*) is the sum of the potential of all possible cliques *V*_*c*_*U*(*X*).

One clique *c* is a subset of location set *S*. In MRF, voxels are related to one another at location set *S* through neighboring system. The neighboring system is defined as *N* = {*N*(**v**_*i*_), **v**_*i*_ ∈ *S*}, where *N*(**v**_*i*_) is the location set of neighboring voxels of voxel **v**_*i*_. The neighboring system adheres to the following rule: **v**_*i*_ ∉ *N*(**v**_*i*_) and **v**_*i*_ ∈ *N*(**v**_*j*_) ⇔ **v**_*j*_ ∈ *N*(**v**_*i*_), where *j* and *i* are the indices of voxels.

The status of one voxel only relates to its neighboring system in the MRF not in all fields (Geman and Geman, 1984). In the neighboring system, the clique potential is defined by a pair of neighboring voxel as follows: *V*_*c*_(*x*(**v**_*i*_), *x*(**v**_*j*_)) = 0.5(1 − *I*_*x*(**v**_*i*_),*x*(**v**_*j*_)_), where *I*_*x*(**v**_*i*_),*x*(**v**_*j*_)_ = 0 if *x*(**v**_*i*_) ≠ *x*(**v**_*j*_); *I*_*x*(**v**_*i*_),*x*(**v**_*j*_)_ = 1 if *x*(**v**_*i*_) = *x*(**v**_*j*_). Additional details about MRF, Gibbs distribution, and clique can be found in the study of Geman (Geman and Geman, 1984).

The computation of the joint probability *p*(Y|X, Θ) is described as follows:

(3) $$\begin{array}{l} p\left(\text{Y}|\text{X},\Theta \right)=\underset{{\text{v}}_{i}}{\prod }p\left(y\left({\text{v}}_{i}\right)|x\left({\text{v}}_{i}\right),\theta_{x\left({\text{v}}_{i}\right)}\right) \end{array}$$

where *p*(*y* (**v**_*i*_)|*x*(**v**_*i*_), θ_*x*(**v**_*i*_)_) is the Gaussian distribution with parameter θ_*x*(**v**_*i*_)_ = (μ_*x*(**v**_*i*_)_, σ_*x*(**v**_*i*_)_). Accordingly, *p*(*y* (**v**_*i*_)|*x*(**v**_*i*_), θ_*x*(**v**_*i*_)_) is the probability computed from *x*(**v**_*i*_) through Gaussian distribution with mean μ_*x*(**v**_*i*_)_ and variance σ_*x*(**v**_*i*_)_. Θ is the set of parameters θ_*x*(**v**_*i*_)_, Θ = {θ_*l*_|*l* ∈ **L**}. Given that *l* is a function that assigns to each voxel a label, θ_*x*(**v**_*i*_)_ = (μ_*x*(**v**_*i*_)_, σ_*x*(**v**_*i*_)_) can also be re-expressed as θ_*l*(*x*(**v**_*i*_))_ = (μ_*l*(*x*(**v**_*i*_))_, σ_*l*(*x*(**v**_*i*_))_), θ_*l*(*x*(**v**_*i*_))_ is the parameter of label *l*(*x*(**v**_*i*_)). Therefore, the joint probability can be further calculated as follows:

(4) $$\begin{array}{l} p\left(\text{Y}|\text{X},\Theta \right)=\underset{{\text{v}}_{i}}{\prod }p\left(y\left({\text{v}}_{i}\right)|x\left({\text{v}}_{i}\right),\theta_{x\left({\text{v}}_{i}\right)}\right)=\underset{{\text{v}}_{i}}{\prod }G\left(y\left({\text{v}}_{i}\right);\theta_{l}\right) \end{array}$$

where *G*(*y* (**v**_*i*_);θ_*l*_) is the Gaussian distribution with parameters, and it can be defined as follows:

(5) $$\begin{array}{l} G\left(y\left({\text{v}}_{i}\right);\theta_{l}\right)=\frac{1}{\sqrt{2\pi \sigma_{l}^{2}}}\exp\left(-\frac{{\left(y\left({\text{v}}_{i}\right)-\mu_{l}\right)}^{2}}{2\sigma_{l}^{2}}\right) \end{array}$$

The expectation–maximization (EM) method (Dempster et al., 1977) is applied to estimate the parameters θ_*l*_ = (μ_*l*_, σ_*l*_). The main parts of the EM method are the E and M steps. The parameters are initialized, thereby resulting in Θ^(0)^. The formula of E step can be defined as follows:

(6) $$\begin{array}{l} \begin{array}{l} Q\left(\Theta |\Theta^{\left(t\right)}\right)=E\left[\log p\left(\text{X},\text{Y}|\Theta \right)|Y,\Theta^{\left(t\right)}\right]\\ =\underset{X\in \chi }{\sum }p\left(\text{X},\text{Y}|\Theta^{\left(t\right)}\right)logP\left(\text{X},\text{Y}|\Theta \right) \end{array} \end{array}$$

where *t* is the index of iterations; Θ^(*t*)^ is the parameter at the *t*-th iteration; *E*[log *p*(**X, Y**|Θ)|**Y**, Θ^(*t*)^] is the expectation of the [log *p*(**X, Y**|Θ)|**Y**, Θ^(*t*)^]; χ is all the possible configurations of the labels. The formula of the M step is as follows:

(7) $$\begin{array}{l} \Theta^{\left(t+1\right)}=\underset{\Theta }{arg\;max}Q\left(\Theta |\Theta^{\left(t\right)}\right) \end{array}$$

When ||Θ^(*t*+1)^ − Θ^(*t*)^|| is smaller than ε or when *t* equals to the maximum iteration, the iterations are stopped.

We can compute the probability images in accordance with the optimal parameters generated from the HMRF–EM method. We take the probability image of GM as an example to explain how we obtain the probabilities. When *l* = *L*_GM_, we obtain θ_*L*_GM__ = (μ_*L*_GM__, σ_*L*_GM__). With the given voxel **v**_*i*_, the joint probability *p*(*y* (**v**_*i*_)|*x*(**v**_*i*_), θ_*x*(**v**_*i*_)_) is computed as follows:

(8) $$\begin{array}{l} p\left(y\left({\text{v}}_{i}\right)|x\left({\text{v}}_{i}\right),\theta_{x\left({\text{v}}_{i}\right)}\right)=G\left(y\left({\text{v}}_{i}\right);\theta_{L_{\text{GM}}}\right)\\ =\frac{1}{\sqrt{2\pi \sigma_{L_{\text{GM}}}^{2}}}\exp\left(-\frac{{\left(y\left({\text{v}}_{i}\right)-\mu_{L_{\text{GM}}}\right)}^{2}}{2\sigma_{L_{\text{GM}}}^{2}}\right) \end{array}$$

The prior probability on the probability image of GM is:

(9) $$\begin{array}{l} p\left(x\left({\text{v}}_{i}\right)\right)=p\left(L_{\text{GM}}\right)\;=\;\frac{1}{Z}\exp\left(-U\left(L_{\text{GM}}\right)\right)\\ =\frac{1}{Z}\exp\left(-\underset{c\in C}{\sum }V_{c}\left(L_{\text{GM}}\right)\right) \end{array}$$

where *Z* is a normalized coefficient (as mentioned in where the MRF is first introduced in this study) and expressed as *Z* = exp(−U(*L*_*GM*_)).

The MRF is considered in the computation of the prior probability, indicating that the value of one voxel is related to the neighboring voxel. This condition provides the final result image with improved consistency. With the given **v**_*i*_, the computation of *p*_GM_ (**v**_*i*_) is:

(10) $$\begin{array}{l} p_{\text{GM}}\left({\text{v}}_{i}\right)=p\left(y\left({\text{v}}_{i}\right)|x\left({\text{v}}_{i}\right),\theta_{x\left({\text{v}}_{i}\right)}\right)p\left(x\left({\text{v}}_{i}\right)\right)\frac{1}{Z\sqrt{2\pi \sigma_{L_{\text{GM}}}^{2}}}\exp\\ \left(-\frac{{\left(y\left({\text{v}}_{i}\right)-\mu_{L_{\text{GM}}}\right)}^{2}}{2\sigma_{L_{\text{GM}}}^{2}}-\underset{c\in C}{\sum }V_{c}\left(L_{\text{GM}}\right)\right) \end{array}$$

The computation of *p*_WM_ (**v**_*i*_) and *p*_*CSF*_ (**v**_*i*_) is similar to the computation of *p*_GM_ (**v**_*i*_). However, *l* = *L*_GM_ needs to be replaced with *l* = *L*_WM_ and *l* = *L*_CSF_.

**Figure 3:**
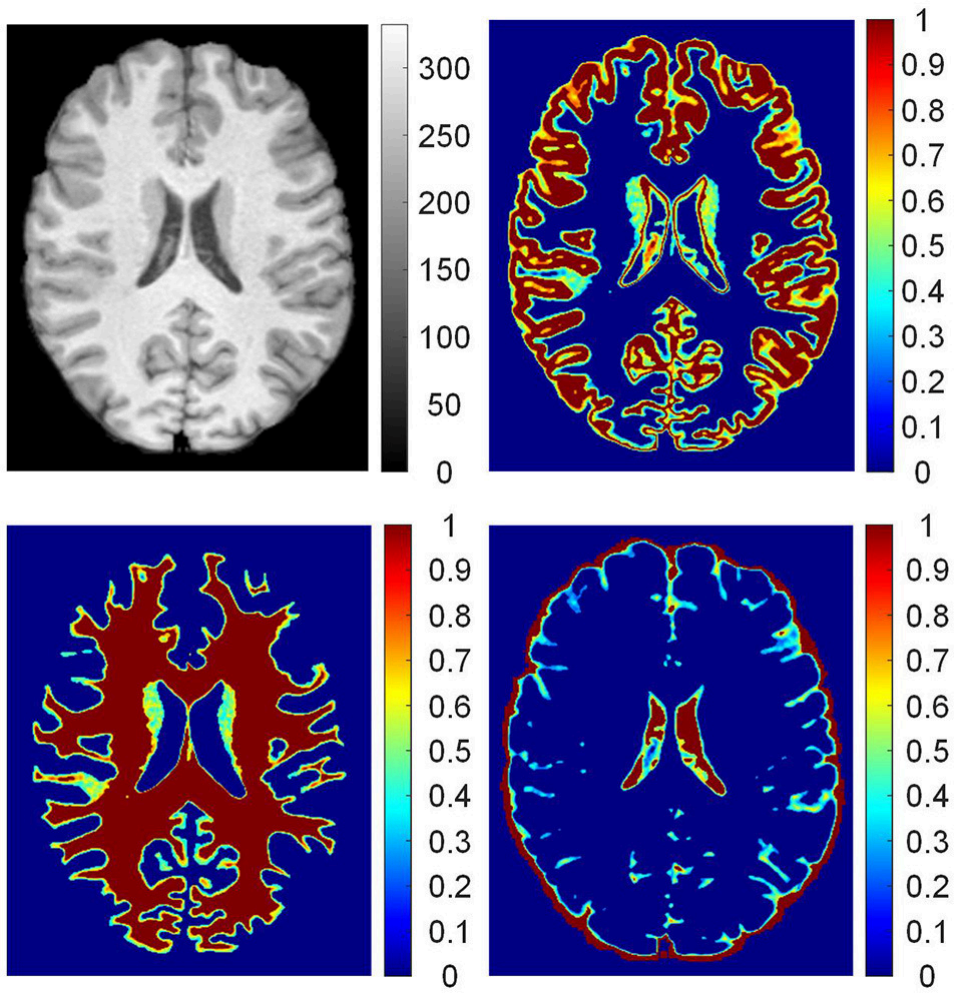
The probability images of brain tissues. From up to down, clockwise, images are: preprocessed T1 weighted magnetic resonance (MR) image; probability images of gray matter (GM), cerebrospinal fluid (CSF), and white matter (WM). On the T1 weighted MR image, values are intensities of the gray image. In the probability images, the values are proportions of brain tissue in the voxels.

The posterior probabilities of each voxel *p*_GM_ (**v**_*i*_), *p*_WM_ (**v**_*i*_), and *p*_*CSF*_ (**v**_*i*_) form three probability images, namely, **P**_GM_, **P**_WM_, and **P**_CSF_. The probability images are shown in Figure 3. The value of each voxel ranges between 0 and 1, representing the probability of the voxel belonging to the brain tissue. For example, one voxel having a value of 0.4 at the probability image of WM can be expressed as *p*_WM_ (**v**_*i*_) = 0.4, which indicates that 40% of the voxel is WM and 60% of the voxel belongs to other brain tissues.

**Figure 4:**
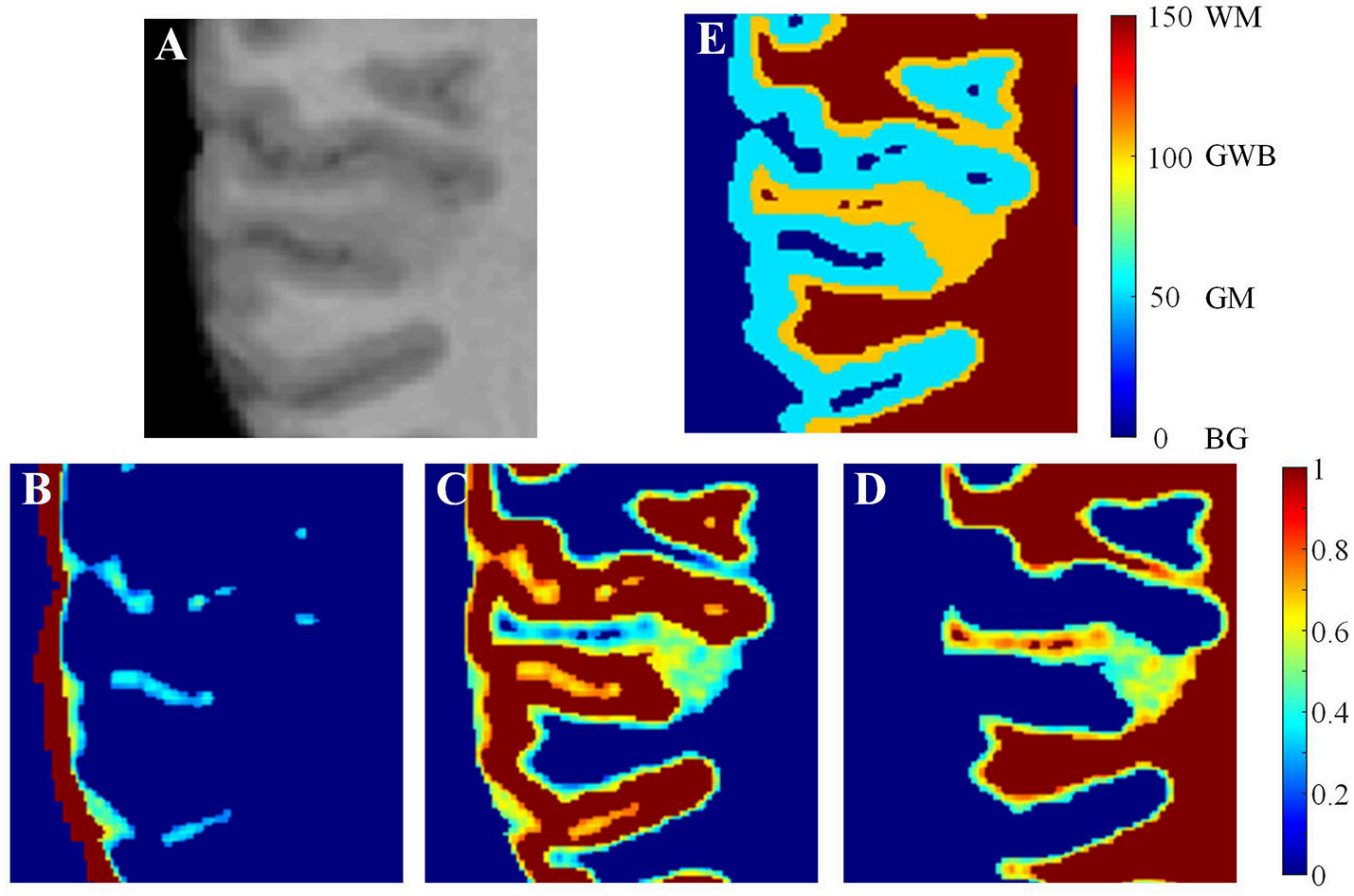
Labeling the regions of interest. In the brain MR image **(A)**, the main tissues are GM, WM, and CSF whose probability images are shown in **(B–D)**. Based on the probability images, the gray/white matter boundary (GWB), white matter (WM), gray matter (GM), and background (BG) are labeled. A small part of an axial slice is enlarged as an example of image taking **(A)**. As a gray scale image cannot present enough detail, we have applied jet color-map to display the images. The image **(E)** is the labeled image with WM, GM, GWB and background according to the probability images.

The procedures for labeling regions of interest are illustrated in Figure 4. On the brain MR images (Figure 4A), the main tissues are GM (Figure 4C), WM (Figure 4D), and CSF (Figure 4B). The regions of interest are labeled according to the probability images to analyze the GWB (Figure 4E), as defined below:

(11) $$\begin{array}{l} L\left(v_{i}\right)=\{\begin{array}{l} L_{\text{GWB}},\\ L_{\text{GM}},\\ L_{\text{WM}}, \end{array}\begin{array}{l} 0<p_{\text{GM}}\left(v_{i}\right)<T_{\text{prob}}\\ \text{ and 0}<p_{\text{WM}}\left(v_{i}\right)<T_{\text{prob}}\\ p_{\text{GM}}\left(v_{i}\right)\geq T_{\text{prob}}\\ p_{\text{WM}}\left(v_{i}\right)\geq T_{\text{prob}} \end{array} \end{array}$$

where *L*(**v**_*i*_) is the label of the voxel **v**_*i*_, *p*_GM_ (**v**_*i*_) is the value of voxel **v**_*i*_ at the probability image of GM, *p*_WM_ (**v**_*i*_) is the value at the probability image of WM, and *T*_prob_ is a threshold to label the probability images and is experimentally set to 0.9. *L*_GM_, *L*_WM_, and *L*_GWB_ are the labels of GM, WM, and GWB, respectively.

#### Local direction for the generation of paths from GM to WM

The local direction described the path from GM to WM for each voxel in the GWB region. The GWB region was subsequently considered as the electric potential field, as described previously (Jones et al., 2000), to generate local directions. The local directions were obtained through iteratively solving the Laplace equation in the GWB region, where the GM and WM were the borders of the electric potential field. The details for generating the local directions are given below.

**Figure 5:**
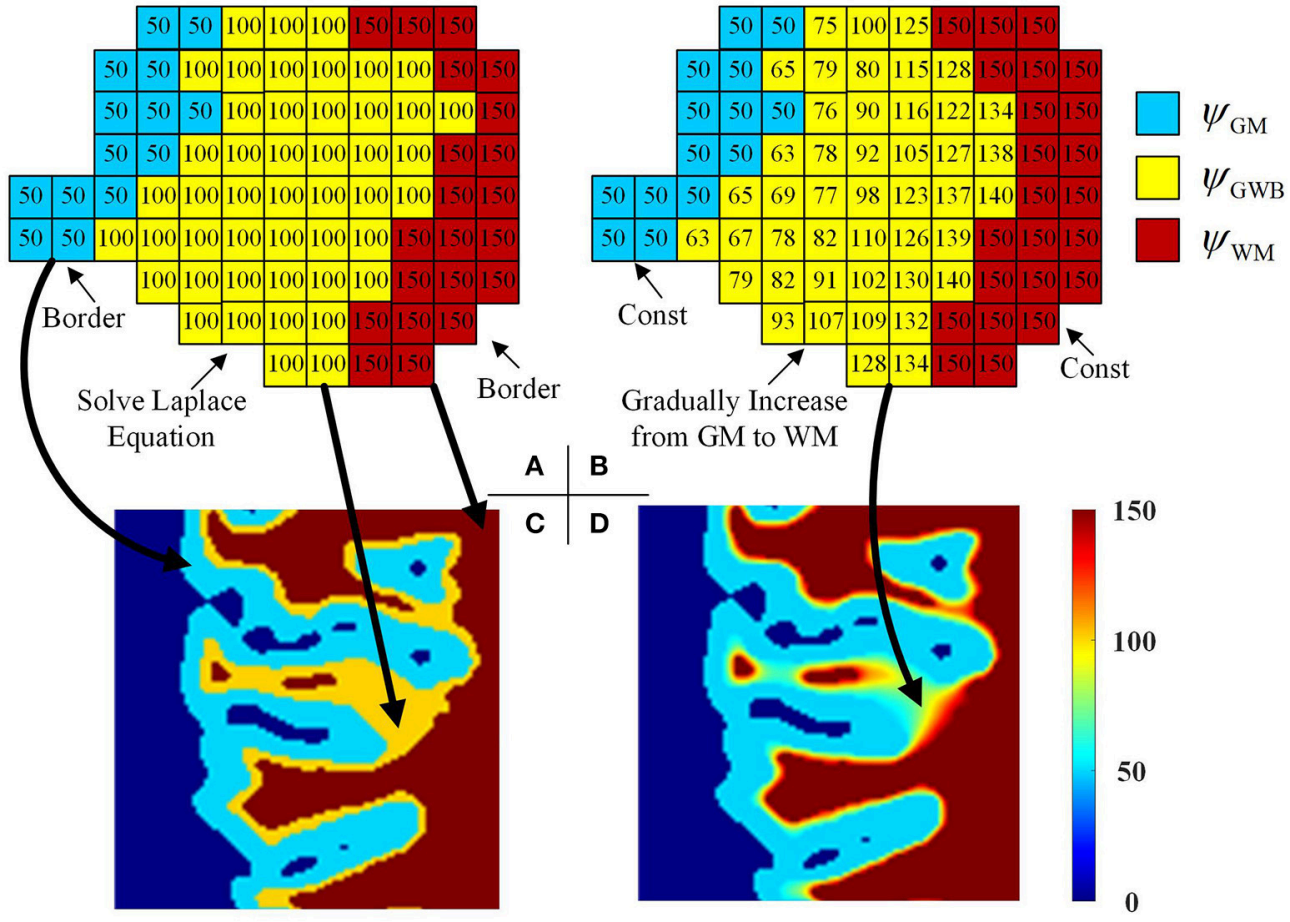
Images illustrating the generation of the local directions from the labeled image. In the labeled image **(A)**, the values on GWB should be larger than that on GM and smaller than that on WM, e.g., 50 on GM, 100 on GWB and 150 on WM. After obtaining the final local direction images **(B)**, the values within GWB gradually increase from GM to WM, while the values within WM and GM keep const. The example enlarged parts of the real labeled image and the real local direction image are shown as in image **(C,D)**, respectively. All computations are in three dimension, for illustrational convenience, we use two dimensional image to explain the method.

Figure 5 illustrates the process of generating the local directions from the labeled image. The GWB region was considered as an electric potential field (Jones et al., 2000). In this field, the value of each point corresponded to the negative gradient of the electric potential value Ψ. The electric potential value was related to the distance between the given point and the border of the electric potential field. In our application, the GM and WM were considered as the two borders of the electric potential field, and the local directions were obtained through solving the Laplace equation within the GWB region.

The Laplace equation in the GWB region was as follows:

(12) $$\begin{array}{l} \nabla^{\text{2}}\psi =\frac{\partial^{2}\psi }{\partial x^{2}}\text{+}\frac{\partial^{2}\psi }{\partial y^{2}}\text{+}\frac{\partial^{2}\psi }{\partial z^{2}}=0 \end{array}$$

where x, *y*, and *z* were the three dimensions in the three-dimensional image, and $\frac{\partial^{2}\psi }{\partial x^{2}}$ was the second-order partial derivative. The potential values of the GM, WM, and CSF were initialized to ψ_GM_, ψ_GWB_, and ψ_WM_, respectively, e.g., ψ_GM_ = 50, ψ_GWB_ = 100, and ψ_WM_ = 150, as shown in Figures 5A,B. The initialized values must obey the rule ψ_GM_ < ψ_GWB_ < ψ_WM_ to guarantee that every voxel within the GWB region has a path from GM to WM. The boundary conditions are as follows: ψ_GM_ = 50 and ψ_WM_ = 150. The values on the GM and WM were unchanged, while the values on the GWB varied on solving the Laplace equation.

The simplest solution to solve the Laplace equation is the Jacobian solution (Jones et al., 2000); we then obtain:

(13) $$\begin{array}{l} \psi_{t+1}\left(\text{x},y,z\right)=[\psi_{t}\left(x+\Delta \text{x},y,z\right)+\psi_{t}\left(x-\Delta \text{x},y,z\right)\\ \;+\psi_{t}\left(x,y+\Delta \text{y},z\right)+\psi_{t}\left(x,y-\Delta \text{y},z\right)\\ \;+\psi_{t}\left(x,y,z+\Delta \text{z}\right)+\psi_{t}\left(x,y,z-\Delta \text{z}\right)]/6 \end{array}$$

where *t* is the index of the iteration. The total electric field energy at the *t*-th iteration is:

(14) $$\begin{array}{l} \varepsilon_{t}={\sum \left[{\left(\Delta \psi_{t}/\Delta \text{x}\right)}^{2}+{\left(\Delta \psi_{t}/\Delta \text{y}\right)}^{2}+{\left(\Delta \psi_{t}/\Delta \text{z}\right)}^{2}\right]}^{1/2} \end{array}$$

when *t* achieves the maximum value, or the condition $\left(\varepsilon_{t+1}-\varepsilon_{t}\right)/\varepsilon_{t}<10^{-5}$ is achieved, the iteration is stopped. Here, the Δψ_*t*_ is obtained through measuring differences between two voxels, e.g., Δψ_*t*_/Δ*x* = [ψ_*t*_(*x* + Δ*x, y, z*) − ψ_*t*_(*x* − Δ*x, y, z*)]/2. The example result after the iterations is shown in Figures 5B,D. Comparison of Figures 5A,B indicates that the potential values in GM and WM regions remain unchanged, whereas the values in the GWB region vary from ψ_GM_ to ψ_WM_.

#### Local directional optimization for GWB width estimation

**Figure 6:**
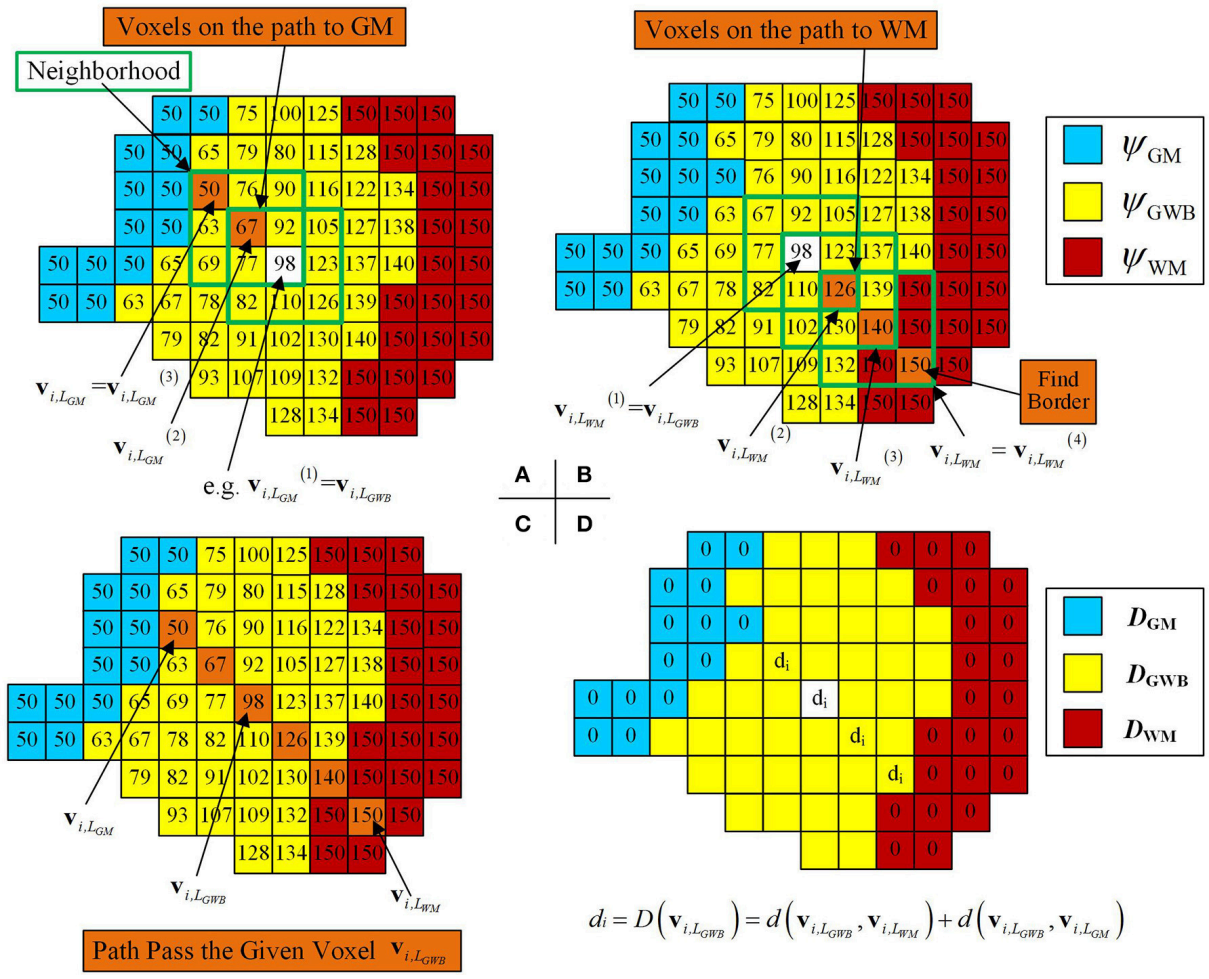
Images illustrating how the optimal local directions were obtained for one example voxel within the GWB, and width computation at the given voxel v_*i,LGWB*_. **(A,B)** Method to find the corresponding voxels on the GM (**v**_*i,LGM*_) and the WM (**v**_*i,LWM*_), through iteratively searching the neighborhood and finding the voxels with smallest and largest potential values. (**C)** An illustrative path from GM to WM for the given voxel **v**_*i,LGWB*_. **(D)** The way to compute the width value at **v**_*i,LGWB*_, according to its **v**_*i,LGM*_ and **v**_*i,LWM*_. For the rest of the voxels within GWB (with yellow base color in **D**), the width values can be obtained by the following procedures.

Local directional optimization aimed to find the corresponding voxels on the GM and WM for each voxel on the GWB to compute the width value of the gray/white matter junction based on the optimal directions. The computational procedure is illustrated in Figure 6. We took one voxel on the local directional image as an example to explain how we computed the width on the voxel. The widths of the remaining voxels were computed the same way. The *i*-th voxel located in the GWB region was denoted by **v**_*i*,__*L*_*GWB*__, e.g., the voxel with the value 98 and a white base color in Figure 6A. The closest voxels to the **v**_*i*,__*L*_*GWB*__ located on GM and WM were denoted by **v**_*i*,__*L*_*GM*__ and **v**_*i*,__*L*_*WM*__, respectively. The estimation of **v**_*i*,__*L*_*GM*__ and **v**_*i*,__*L*_*WM*__ was based on the following equation:

(15) $$\begin{array}{l} {v_{i,L_{GM}}}^{\left(t+1\right)}=\underset{q\left(t\right)\in N\left({v_{i,L_{GM}}}^{\left(t\right)}\right)}{arg\;max}\psi \left(q^{\left(t\right)}\right) \end{array}$$

where *t* ≥ 1 was the index of iteration, ${v_{i,L_{GM}}}^{\left(t+1\right)}$ was the center voxel for finding **v**_*i*,__*L*_*GM*__ at the (*t* + 1)-th iteration, $N\left({v_{i,L_{GM}}}^{\left(t\right)}\right)$ was the local window of ${v_{i,L_{GM}}}^{\left(t\right)}$, and **q**^(*t*)^ was the neighboring voxel of ${v_{i,L_{GM}}}^{\left(t\right)}$ within $N\left({v_{i,L_{GM}}}^{\left(t\right)}\right)$. The iteration was stopped when $\psi \left({v_{i,L_{GM}}}^{\left(t\right)}\right)=\psi GM$, and we obtained $v_{i,L_{GM}}={v_{i,L_{GM}}}^{\left(t\right)}$. The initialization was ${v_{i,L_{GM}}}^{\left(1\right)}=v_{i,L_{GMB}}$.

In Figure 6A, the voxel with a predefined value and white base color ${v_{i,L_{GM}}}^{\left(1\right)}$ within the green square was considered to be the center voxel during the first search *t* = 1. On searching the vicinity of ${v_{i,L_{GM}}}^{\left(1\right)}$, the voxel with the value 67 was considered to be ${v_{i,L_{GM}}}^{\left(2\right)}$ because $\psi \left({v_{i,L_{GM}}}^{\left(2\right)}\right)$ had the smallest value among ψ(*q*^(*t*)^) in the local directional image. Similarly, ${v_{i,L_{GM}}}^{\left(3\right)}$ was the voxel with the value 50 (example values of ψ_GM_ = 50, ψ_GWB_ = 100, and ψ_WM_ = 150, as mentioned in Equation 12) within the green square, where $\psi \left({v_{i,L_{GM}}}^{\left(3\right)}\right)=\psi_{GM}$. Therefore, ${v_{i,L_{GM}}}^{\left(3\right)}$ was the **v**_*i*,__*L*_*GM*__ because the voxel with the value 50 equaled the value in the GM region, denoted by $v_{i,L_{GM}}={v_{i,L_{GM}}}^{\left(3\right)}$.

Similarly, **v**_*i*,__*L*_*WM*__ was derived based on the following equation:

(16) $$\begin{array}{l} {v_{i,L_{WM}}}^{\left(t+1\right)}=\underset{q\left(t\right)\in N\left({v_{i,L_{WM}}}^{\left(t\right)}\right)}{arg\;max}\psi \left(q^{\left(t\right)}\right) \end{array}$$

where ${v_{i,L_{WM}}}^{\left(t+1\right)}$ was the center voxel for finding **v**_*i*,__*L*_*WM*__ at the (*t* + 1)-th iteration. When $\psi \left({v_{i,L_{GWB}}}^{\left(t+1\right)}\right)=\psi WM$, the iteration was halted, and we obtained $v_{i,L_{WM}}={v_{i,L_{GWB}}}^{\left(t\right)}$.

In Figure 6B, the path from the given voxel ${v_{i,L_{WM}}}^{\left(1\right)}$ to the WM (where ${v_{i,L_{WM}}}^{\left(1\right)}=v_{i,L_{GWB}}$) was composed of voxels with orange base color and within the green square. The potential value of ${v_{i,L_{WM}}}^{\left(2\right)}$ = 126, $\psi \left({v_{i,L_{WM}}}^{\left(3\right)}\right)=140$, and $\psi \left({v_{i,L_{WM}}}^{\left(4\right)}\right)=150$ on the illustrative image of Figure 6B.

All voxels, which were found on searching for **v**_*i*,__*L*_*WM*__ and **v**_*i*,__*L*_*GM*__ for the given voxel **v**_*i*,__*L*_*GWB*__, formed a path from the GM to WM passing through the voxel **v**_*i*,__*L*_*GWB*__, as shown in Figure 6C. The widths for these voxels on the path were related to the locations of **v**_*i*,__*L*_*WM*__, **v**_*i*,__*L*_*GM*__, and **v**_*i*,__*L*_*GWB*__. The GWB width at the voxel *v*_*i*,__*L*_*GWB*__ was computed using Equation (1).

On computing all the widths for the voxels in the GWB region, images with the widths were obtained and regarded as the GWB width map, denoted by **D** = (*D*(**v**_1_), …, *D*(**v**_*i*_), …, *D*(**v**_*N*_)). In this way, these width values were not computed using voxels outside the GWB region, where *D*(**v**_*i*_) = 0. In the GWB width map, the regions with large values indicated possible FCD lesions with blurred GWB feature. The procedure for computing the GWB width map from the input T1-weighted image has been summarized in Table 1.

**Table 1:** The computational procedure of the gray/white matter boundary (GWB) width map.

**Method**: LDPO Method
**Input:** Gray Scale Image **Y**
**Output:** GWB Width Map **D**
Segment *Y* and find the parameters of GM, WM and CSF according to Equation (7) (Section Preprocessing). Compute probability images of GM, WM and CSF according to Equation (10) (Section Preprocessing). Label regions of interest according to Equation (11) (Section LDPO for GWB Width Estimation). Generate local directions through solving Laplace Equation on GWB region according to Equation (13) (Section LDPO for GWB Width Estimation). Find the optimal corresponding voxels in WM and GM for each voxel on GWB according to Equation (15) and Equation (16) (Section Evaluations). Measure the width (Equation 1) according to the optimal corresponding voxels in WM and GM (Section Datasets and Ground Truth).

### Evaluations

The brain MR images of 10 patients and 31 healthy individuals were studied to validate the effectiveness of the proposed method. For fair comparison, all features were evaluated by the same method. The evaluation methods included the receiver operating characteristic (ROC) curve analysis of true positive rate (TPR), false positive rate (FPR), F-score, relations of precision, and recall. These values were computed from the true positive (TP), true negative (TN), false positive (FP), and false negative (FN).

For the ROC curve analysis, the evaluation step was as follows. On the three-dimensional GWB width map of each patient, the voxel with a value greater than *T* was considered positive. *T* varied from the minimum to the maximum value in the GWB width map. The image was evaluated using the voxels, labeled as negative or positive, by comparing with the ground truth; this resulted in TP, TN, FP, and FN. The FPR, TPR, precision, and recall were computed from TP, TN, FP, and FN.

We evaluated the different features using F-scores for all patients in this study. As the lesional region was much smaller than the whole brain, we randomly selected axial slices with lesions to compute the F-score, for effective evaluation of different features. We applied the same method of evaluation for each feature. For each feature with a threshold value *T*, we computed the values of F-score, and the value was a point on the figure of F-score. For the GWB width, cortical thickness, and relative intensity maps, voxels with values greater than *T* were considered positive. For the gradient map, voxels with values less than *T* were considered positive. Moreover, for the gradient map, voxels with values less than 5 were considered as background and not included in the evaluations.

## Experimental results

**Figure 7:**
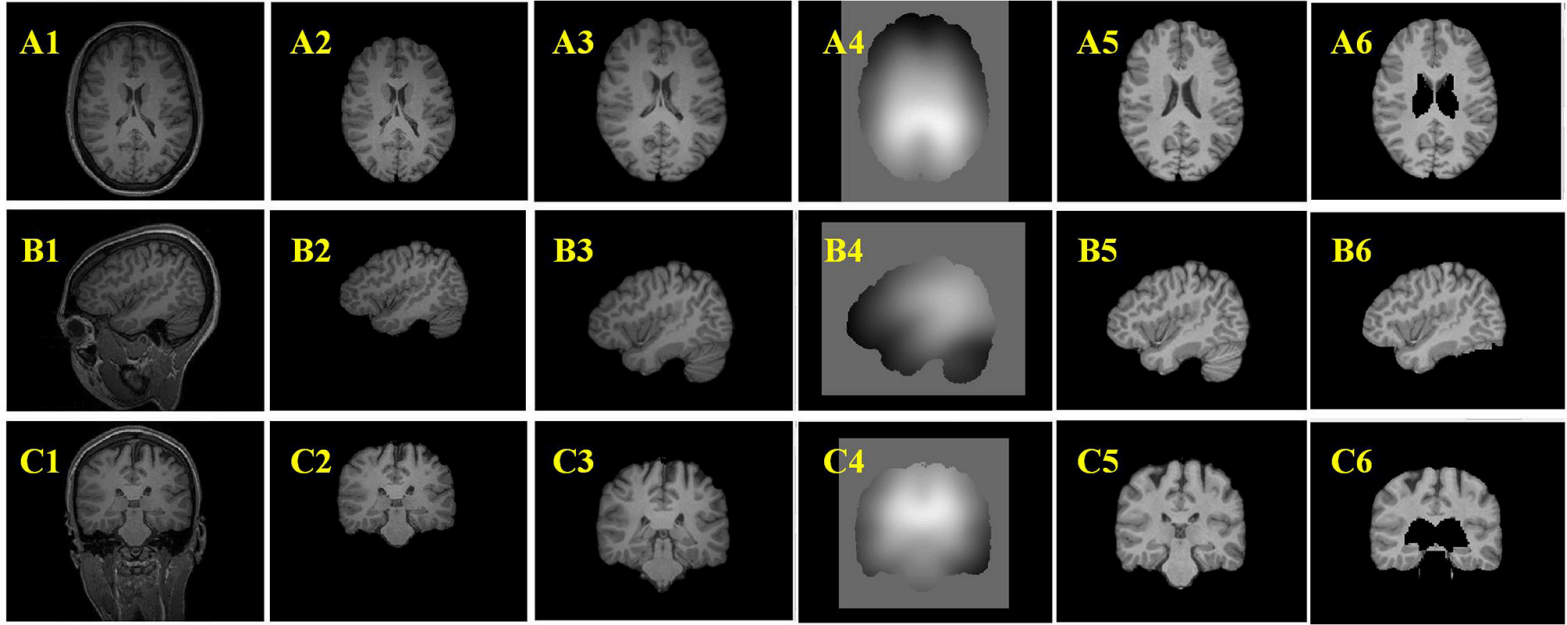
Example results of the preprocessing procedures. From up to down, the three rows are images of axial view **(A)**, sagittal view **(B)**, and coronal view **(C)**. From left to right, the columns are original images **(A1–C1)**, images after brain extraction **(A2–C2)**, images after registered to Montreal Neurological Institute (MNI) brain space **(A3–C3)**, bias field images **(A4–C4)**, bias field corrected images **(A5–C5)**, images after removing tissues which are not related to FCD lesions **(A6–C6)**.

**Figure 8:**
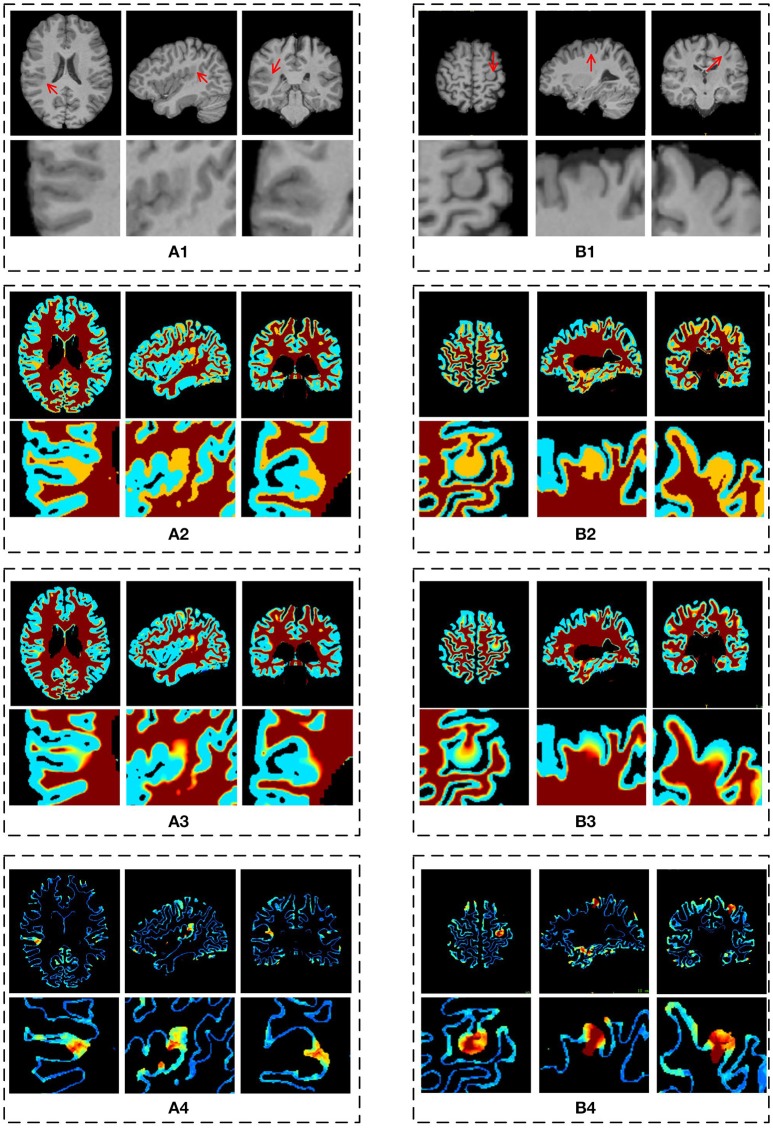
The results of different stages on GWB width map computation. Images are from two participants denoted by **(A,B)**, respectively. The **(A1,B1)** are the preprocessed images with lesional regions indicating by red arrows. The **(A2,B2)** are segmentation images with GWB region. The **(A3,B3)** are the images with local directions. The **(A4,B4)** are the GWB width maps. On each dashed box, from left to right, the columns are the image on axial, sagittal, and coronal views. The images on the second line show the enlarged FCD regions [the locations are indicated by red arrows on the first line of **(A1,B1)**] on the corresponding image on the first line. All the computations are three-dimensional; for convenience, two-dimensional images in different views are presented.

**Figure 9:**
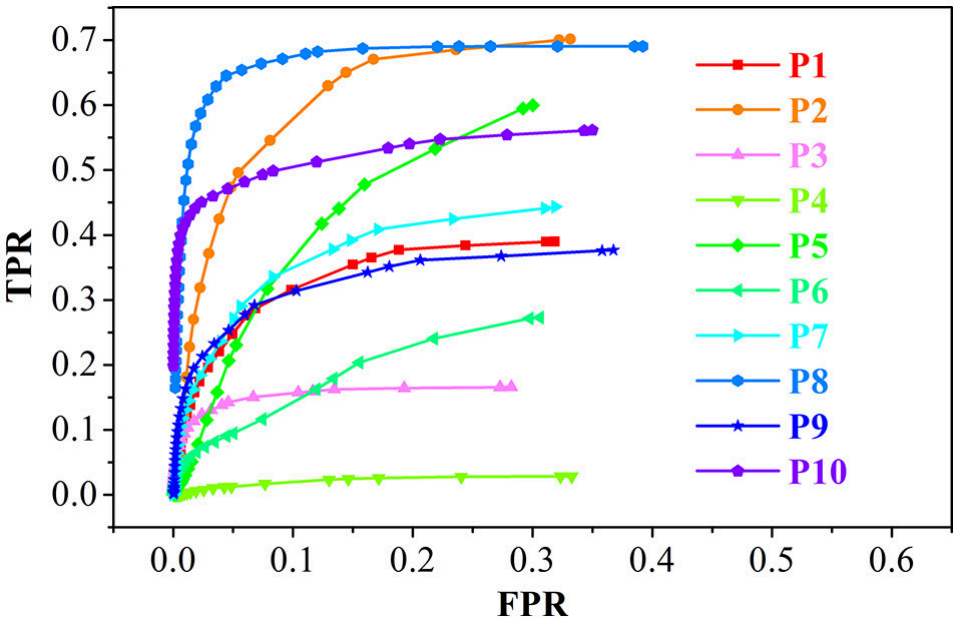
The receiver operating characteristic (ROC) curve analysis of the true positive rate (TPR) and false positive rate (FPR) for the feature images of the GWB width map. P1 means the results of patient number 1.

**Figure 10:**
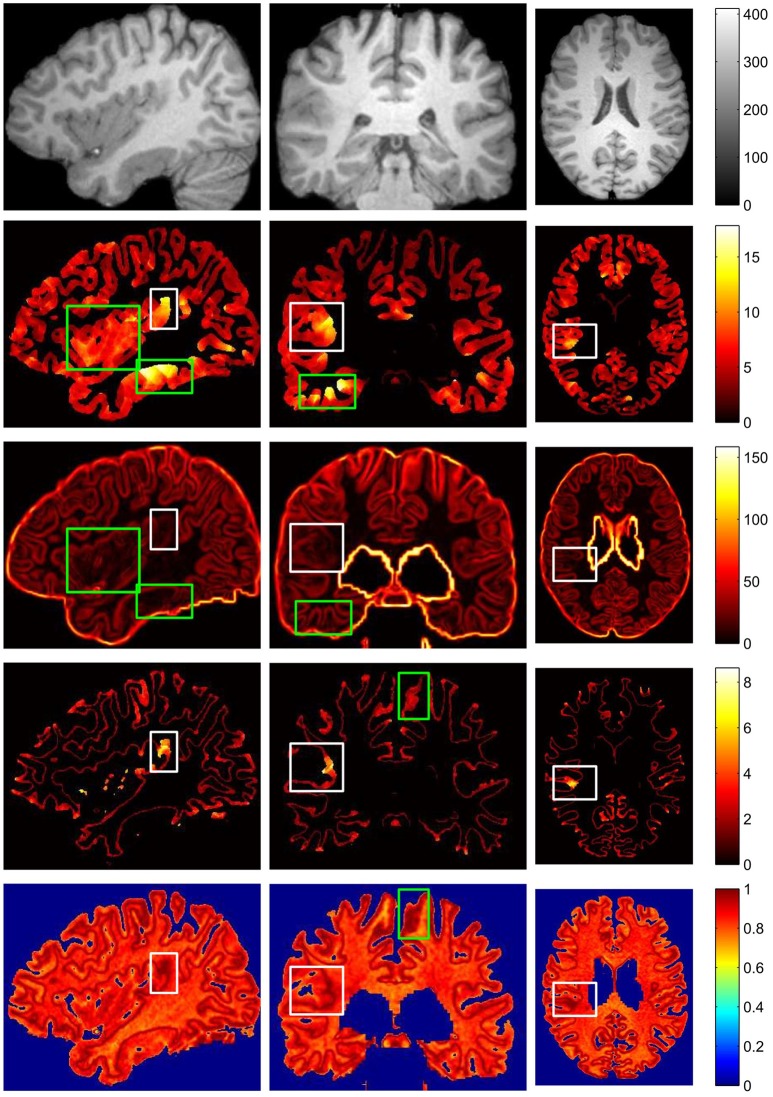
The comparison of different FCD feature maps in three views. From up to down, images on each row are preprocessed MR image, gray matter or cortical thickness map, gradient map, GWB width map, and relative intensity map. From left to right, columns are sagittal view, coronal view and axial view. The images are randomly extracted from three dimensional image from one patient. The true FCD lesional regions are surrounded by white rectangles, while the false positive regions are indicated by green rectangles.

**Figure 11:**
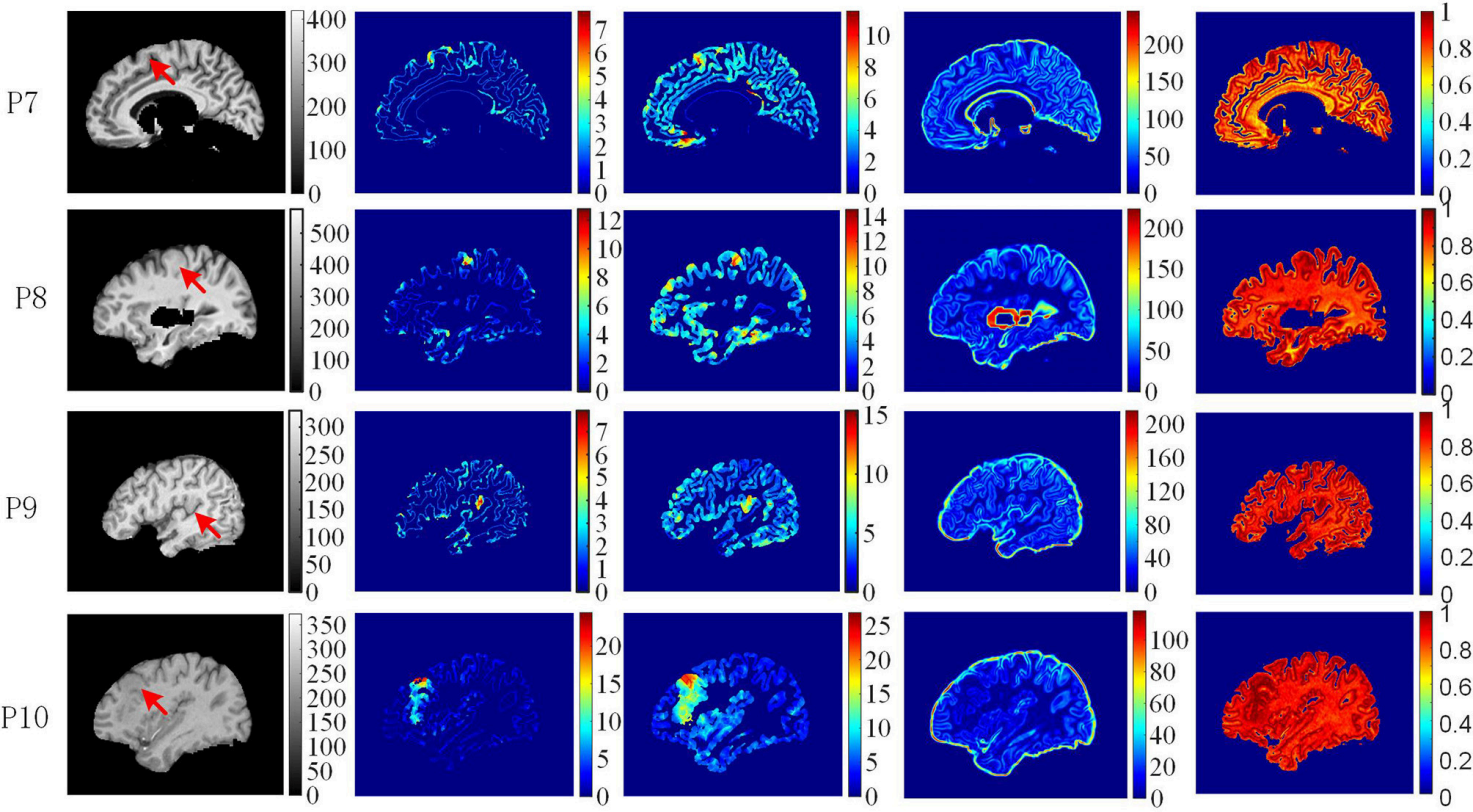
The comparison of feature maps in different patients. Each row shows the same image slices of one example patient. From left to right, columns are preprocessed MR images, GWB width map (unit is mm), the cortical thickness map (unit is mm), the gradient map and the relative intensity map. On the preprocessed images, the lesional regions are indicated by red arrows.

**Figure 12:**
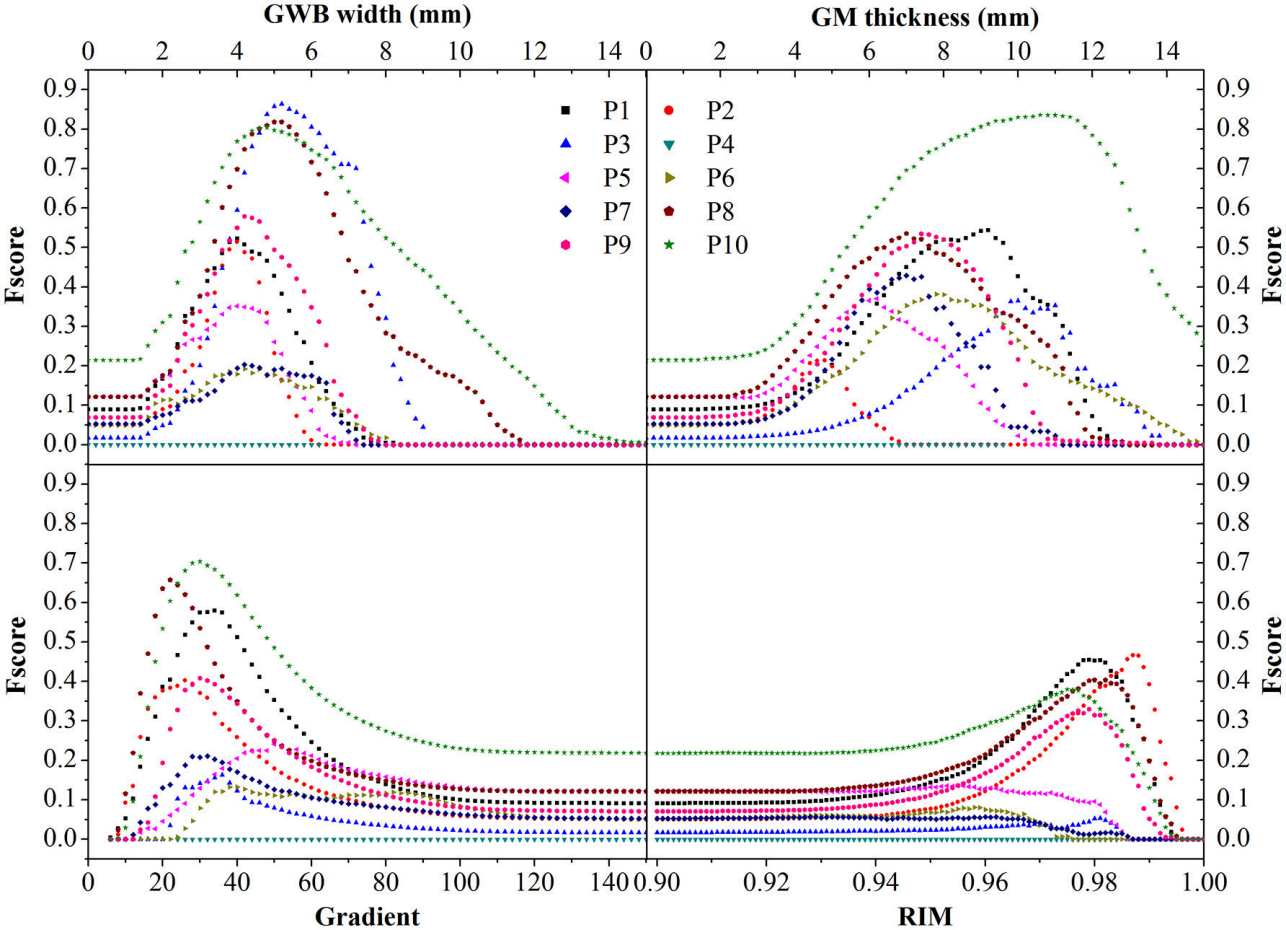
The comparison in terms of F-score of FCD features. The feature maps are GWB width map, gray matter (GM) thickness map, gradient map, and relative intensity map (RIM). Generally, the proposed GWB width map could achieve higher F-score values indicating better performances than the other feature maps.

**Figure 13:**
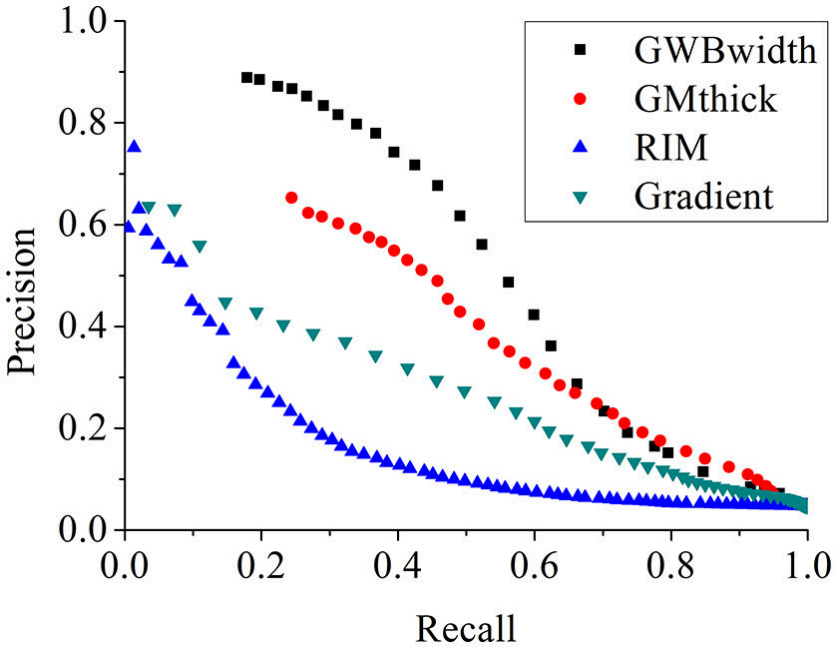
The comparison of FCD feature maps using precision and recall. The feature maps are the same as those on Figure 12. The proposed GWB width map produces better trade-off between precision and recall in terms of detecting the blurring of junction between GM and WM.

The example results of the preprocessing are presented in Figure 7. The results of different stages of the GWB width map computation are depicted in Figure 8. Table 2 summarizes the statistical values of the FCD lesional and non-lesional regions of patients and the GWB region of healthy controls on the GWB width map. Figure 9 presents the evaluation of the proposed GWB width map in terms of TPR and FPR. Figures 10–**13** compare the results of the GWB width map with those of other widely applied FCD features, including gradient map, relative intensity map, and GM thickness map.

**Table 2:** The statistical values on the GWB map of the regions with and without focal cortical dysplasia (FCD) in patients, and gray white matter boundary (GWB) in healthy controls.

**PI**	**FCD**			**GWB-FCD**			**HCI**	**GWB of healthy controls**		
	**(Mean ± std)**	**Max/Min**	**Mode**	**(Mean ± std)**	**Max/Min**	**Mode**		**(Mean ± std)**	**Max/Min**	**Mode**
1	3.63 ± 1.28	9.56/1.21	2.28	2.28 ± 0.90	13.02/0.87	1.57	1	2.37 ± 0.96	17.88/0.87	1.57
2	3.30 ± 0.93	6.77/1.37	2.28	2.15 ± 0.79	11.26/0.87	1.57	2	2.35 ± 1.00	17.38/0.71	1.57
3	4.31 ± 1.65	10.07/1.21	3.46	2.10 ± 0.71	9.94/0.71	1.57	3	2.51 ± 1.04	13.35/0.71	1.73
4	2.76 ± 0.73	4.93/1.57	2.37	2.13 ± 0.77	13.89/1.00	1.57	4	2.28 ±0.84	15.23/0.71	1.57
5	2.59 ± 0.88	8.63/ 1.21	1.57	2.18 ± 0.82	12.44/0.71	1.57	5	2.44 ± 1.00	15.02/0.71	1.73
6	2.77 ± 1.28	8.09/1.37	1.73	2.13 ± 0.75	12.22/0.71	1.57	6	2.36 ± 0.99	14.11/0.71	1.73
7	3.44 ± 1.34	8.15/1.21	2.37	2.18 ± 0.78	13.77/0.71	1.57	7	2.35 ±0.96	16.53/1.00	1.57
8	6.49 ± 2.34	13.55/1.73	5.20	2.49 ± 1.06	16.93/0.71	1.57	8	2.34 ±0.92	13.99/0.87	1.57
9	3.97 ± 1.55	9.74/1.21	2.37	2.20 ±0.77	14.37/1.00	1.57	9	2.26 ±0.88	16.88/0.87	1.57
10	7.02 ± 3.72	24.69/1.21	2.28	2.32 ± 0.81	12.58/0.87	1.57	10	2.27 ±0.94	16.84/0.71	1.57

*Unit of measurement, millimeters. PI, patient index; HCI, healthy controls index*.

Figures 7A2–C2 are the images after brain extraction from the original images in Figures 7A1–C1. The brain extracted images were registered to the MNI T1-weighted MR images in Figures 7A3–C3. The images after bias field correction (Figures 7A4–C4) and intensity normalization are presented in Figures 7A5–C5. Figures 7A6–C6 present the example results after removing the regions that are not related to the FCD lesion. The structures of the GM and WM on the images in Figures 7A5–C5 are more clear compared to that on the images in Figures 7A3–C3. The bias-corrected images provided improved space information of the GM and WM for the subsequent analysis of broadened GWB. The bias field images (Figures 7A4–C4) indicate that the MR images exhibit inhomogeneity of intensity. After bias field correction, the GM and WM regions show coherent intensities. This means that the voxels at different locations belonging to the GM or WM on the bias field images have similar intensities compared to the intensity of the voxels before bias field correction. The central region of Figure 7A5 indicates that deep GM regions can be easily misidentified as FCD lesion due to fact that the intensities and gradients in deep GM are similar to those of the blurred GWB within the FCD lesion. The brain regions that are not related to the FCD lesions should be eliminated from the images to decrease the number of FP results. An earlier study has also reported that the FCD non-related regions should be disregarded when evaluating the results of FCD detection (Antel et al., 2003).

The main processing results of the GWB width map computation are depicted in Figure 8. On the enlarged FCD lesional region of the preprocessed images (second line in Figures 8A1,B1), the blurring of junction between the GM and WM can be seen. As evidenced on the segmented (or labeled) images (Figures 8A2,B2), the FCD lesional region has a wider GWB region; however, the extent of GWB region broadening and its normal width remain unclear. The location directions (Figures 8A3,B3) indicate that the values on the GWB gradually increased from the GM to the WM. On the GWB width map (Figures 8A4,B4), the lesional region had a larger value than the non-lesional region.

Table 2 summarizes the statistical values on the FCD lesional region and non-lesional region (GWB subtracts FCD) of patients, and GWB region of healthy controls for the purpose of quantifying the GWB width. The range of mean GWB width values were 2.59–7.02 mm in the FCD region, 2.10–2.49 mm in the FCD non-related region, and 2.26–2.50 mm in the GWB region of healthy controls. In general, mean GWB width values on the lesional region of patients were larger than those on the non-lesional regions of both patients and healthy controls. Most of the mode values on normal GWB width of patients and GWB width of healthy controls were 1.57 mm. The minimal values in the lesional region varied from 1.2 to 1.37 mm, which is smaller than the mode values in the non-lesional regions.

The minimal values (Table 2) in the lesional region were smaller than the mode values in the non-lesional regions, because the lesional regions are larger than the blurred GWB. This means that the lesional regions include both the blurred and non-blurred GWB regions. These non-blurred GWB regions were located within lesional regions and presented similar width values to the values in the non-lesional region. The maximum values on the three types of regions indicated that no simple threshold could differentiate them. The reason for this may have been because the GWB width values on the whole brain is not uniform; indeed, on some regions such as the bottom of the brain, it is normal for the GWB to be wider.

With respect to the values exhibited on the images of P10 in Table 2, the GWB width in the lesional region was the largest among all patients examined in this study, indicating a largely broadened GWB within the lesional region. This finding is in agreement with the fact that the lesional region of P10 was the largest, as shown in Figure 11. With respect to P5, the mean value was the smallest among all patients. As evidenced by the MR images, P5 did not exhibit blurred or broadened GWB. An earlier study reported that 30% of patients with FCD lesions do not have blurred or broadened GWB feature (Bernasconi and Bernasconi, 2011).

The performance of the proposed LDPO method on different patients in terms of ROC curve analysis, including TPR and FPR is presented in Figure 9. The TPR of P8 was greater than that of the others, whereas the FPR were comparable, indicating that the blurred GWB feature in P8 was the most obvious among all patients in this study. For P4, the TPR was less than 0.05 among all *T* ranges, suggesting that the FCD lesions in P4 did not have a blurred GWB feature. In general, when the lesional region presents with blurring at the junction of the GM and WM, the proposed method gives a good trade-off between TPR and FPR.

The vivid comparison of the proposed GWB width map and the other FCD feature maps are demonstrated in Figure 10. In the cortical thickness map (the second row of Figure 10), the increased cortical thickness feature appeared not only in the lesional region (inside a white rectangle) but also in the temporal lobe and bottom brain regions (inside green rectangles). In the gradient map (the third row of Figure 10), the blurred GWB region (inside a white rectangle) had low gradient values; however, the regions with a large GM size (surrounded by green rectangles) also had low gradient values due to the slow variation in intensities. These green rectangle-surrounded regions were easy to misidentify as positive when using gradient map to detect lesions. In the relative intensity map (RIM; the forth row of Figure 10), the blurred GWB regions had large values and formed darker red regions. Apart from the lesional region (within a white rectangle), the darker red regions were along the GWB, thereby forming a line. The voxels on the line on RIM were prone to be incorrectly identified as positive because they had similar values to those in the true lesional region. In the GWB width map (the fifth row of Figure 10), the lesional regions had larger values than the non-lesional region. On the top brain regions, the GWB width values are large, but these regions did not belong to those in the lesional region. The reason for that may be the large amount of incoming or outgoing fibers in the cortical areas around the central sulcus (Huppertz et al., 2005).

The comparison of feature maps in different patients is presented in Figure 11. On the preprocessed images (the first column from left), the lesional locations (indicated by red arrow) were randomly distributed in the brain regions. In the GWB width maps (the second column), the lesional regions had values ranging from 4 to 20 mm. In the cortical thickness maps (the third column), the values in the lesional regions varied from 8 to 25 mm. In the gradient maps (the fourth column), the lesional regions had small values and merged together with the background regions. In the relative intensity maps (the fifth column), the values in the lesional regions were expected to equal 1. The GWB width map could not only highlight the lesional region with blurred GWB but also quantitatively measured the extent to which the GWB broadened; for example, the widest GWB was 7.5 mm for P9.

The evaluation of different features using F-scores for all the patients (P1–10) in this study are shown in Figure 12. Generally, the proposed GWB width map obtained better (or higher) F-scores than the other FCD features. For example, the F-scores of the GWB width map of three patients (P3, P8, and P10) were 0.8; the F-score of only one patient (P10) was greater than 0.8 for the GM thickness, and the F-scores of gradient map and the RIM were all less than 0.8. For P4, the F-scores of all the methods equaled zero, suggesting that the lesion of P4 was not different from non-lesional regions on the feature maps.

The comparison of different FCD feature maps in terms of precision and recall is demonstrated in Figure 13. The values were the mean evaluation results of all patients on the feature images. The image indicated that, when recall was less than 0.7, the precision of the GWB width map was the greatest among all features; whereas when precision was greater than 0.23, the recall of the GWB width map was the greatest. This indicates that when the precision and recall are modest, the proposed method provides improved trade-off between precision and recall. The GM thickness also outperformed other FCD feature images when the recall was greater than 0.7; in such a situation, the precision is quite small. Therefore, the proposed GWB width map could give better trade-off between precision and recall, as evidenced by the evaluation results of F-scores in Figure 12.

## Discussions

The preprocess stages (Figure 7) improved the image quality and were the critical steps for segmentation of the GWB region. The bias field correction and intensity normalization stages made the intensities of the GM and WM more stable, meaning that though the intensities could not be constant, they varied within a smaller range than before the preprocess stages. The registration step enabled to locate the brain tissues (including both FCD related and FCD non-related regions) through borrowing the atlases of MNI brain. The registration, brain extraction, and elimination of FCD non-related regions aimed for greater accuracy in region of intensity.

The resulting images (Figure 8) of steps in computing the proposed feature map confirmed that the proposed GWB width map could effectively distinguish the lesional and non-lesional regions. Compared to the preprocessed images, the proposed GWB width map highlighted the lesional region, which had blurring of junction or broadened junction between the GM and WM. The variation of the enlarged FCD lesional region clearly validated that the proposed method was able to analyze the lesional region quantitatively.

The statistical results of the GWB width values (Table 2) quantitatively confirmed that the blurring GWB region was broadened and became wider than normal GWB (GWB without FCD lesion in patients and GWB in healthy controls). The mean values in FCD regions varied in a relative big range, indicating that the blurring degrees of GWB were different for different patients. The mean values on non-lesional regions were similar, indicating that the proposed method was effective and robust in modeling the GWB width, because the values were relatively stable on the non-lesional region.

According to the vividly shown results of different FCD features in Figure 10, we can conclude that no feature map is perfect, and that each feature map has regions that are easy to be incorrectly classified as FP. However, these regions may be at different locations in different feature maps. This condition indicates that the FCD lesions should be detected using multiple feature maps instead of only one feature map in the future.

In terms of highlight the blurring of junction between GM and WM, the proposed GWB width map outperformed the other FCD feature maps (Figure 11). In the RIM, the lesional regions were prone to be mixed together with the whole junction of GM and WM. On the gradient map, although the blurred GWB could be differed from normal GWB, the lower values in the blurred GWB were hidden in the background. In addition, the gradient map could not quantitatively measure the extent of the blurred GWB from GM to WM. The GWB width map and the cortical thickness map effectively highlighted the lesional region. However, the cortical thickness map focused on the GM region, while the GWB width map focused on the junction between GM and WM.

The statistical evaluation results on analyzing the blurring of GWB (Figure 12) confirmed that the proposed GWB width map performance was better than the three remaining FCD features. Compared to the gradient map and the RIM, the GWB width map and the GM thickness map produced better (higher) F-score values. The higher F-scores indicated that the GWB width map provided better trade-off between precision and recall, as evidenced by Figure 13. Compared to the GM thickness map, it was easier to find an optimal threshold for achieving highest F-scores with the GWB width map. For example, when threshold *T* was approximately set to 4–5 mm on the GWB width map, the F-score values of P1–10 achieved the highest values. However, the optimal thresholds on the GM thickness map for different patients varied significantly. For P2, when *T* corresponded to 5 mm, the F-score achieved the largest value corresponding to 0.4. For P10, the possible values of *T* were approximately 10–12 mm.

Even the exits of blurring GWB might be not support from neuroscience, the blurring GWB is reported as an important FCD lesional feature on MR images by several studies (Huppertz et al., 2005; Hong et al., 2014; Kini et al., 2016). Kini et al. (2016) demonstrated that these blurred junctions contribute to a pseudo-thickening of the cortex (Kini et al., 2016). Bernasconi and Bernasconi (2011) reported that up to 72–96% of FCD lesions have blurring of the GWB. Kini et al. (2016) have reported that up to 83% of the patients who have FCD lesions, but no imaging findings, had subtle GWB blurring that was initially missed by the neuroradiologist (Kini et al., 2016). Therefore, we believe that the blurring GWB is an important feature of FCD lesions.

The GWB region is located between GM and WM and was segmented according to the intensities and locations in this study. Indeed, partial volume effect is a main reason of intensity change from GM to WM. Moreover, when large amounts of incoming or outgoing fibers exist in a region, the junction between GM and WM also appears to be wider (Huppertz et al., 2005).

The present study has several limitations. First, not all the FCD lesions presented with a blurred GWB. For future automated FCD detection, it is important to apply GWB width map together with other FCD feature maps. Second, GWB widths depend on the segmentation of the GWB region. Different segmentation methods may give slightly different GWB region; thus, the resulting GWB width may slightly vary. Patients and healthy controls must be processed using the same segmentation method in order to use GWB width feature for detection of FCD. In this way, the GWB width of healthy controls can be considered as baseline. Third, it is a challenge to compare the GWB width map between patients and healthy controls. The GWB region is thin, and different persons have different crispation of the cortex and GWB. Therefore, the GWB width values in patients and healthy controls are not comparable voxel by voxel. In future, it is necessary to compare the GWB width values using a local window or apply affine and non-linear registration before comparison. Finally, the number of datasets used in this study is limited. We have made an effort to find publicly available MRI datasets containing FCD lesions. To the best of our knowledge, only one database (http://eeg.pl/epi) of epilepsy patients is available for public research, of which only three cases contained FCD lesions. However, these patients were infants, whose brain tissues were immature; therefore, their images were unsuitable for use in this study. Kini et al. (2016) cited the lack of public FCD datasets as a significant problem in FCD research.

## Conclusions and discussions

In this study, we proposed a novel method to measure the width of the GWB. The GWB width map is utilized to quantify the blurred and broadened GWB. The proposed LDPO method solves the three main issues in computing the GWB width values. In the proposed method, the HMRF-EM approach is first introduced to calculate the probability information of brain tissues in order to obtain the GWB region that is the focus of this study. The local directions on each voxel within the GWB, from GM to WM, are then generated through iteratively solving the Laplace equation in the GWB region. The iterative searching method for obtaining the corresponding voxels on GM and WM has been proposed to find the optimal local direction for each location. Finally, the width on each location is computed on the basis of the optimal local direction.

The proposed method was validated on MR images of 10 real patients with FCD lesions and 31 healthy persons. The existing FCD features, including cortical thickness map, gradient map, and relative intensity map were compared with the proposed GWB width map. The GWB width map could quantify the blurred and broadened GWB, whereas the gradient and relative intensity maps could not quantitatively analyze the blurred GWB. The cortical thickness map mainly focused on the GM region that had constant intensities, whereas the GWB width map focused on the GWB regions with significantly varying intensities. The curves of precision and recall revealed that the GWB width map generated from the proposed LDPO method had better trade-off between precision and recall than the other FCD feature maps. The possible FP regions generally have different locations in different feature maps. Therefore, the proposed GWB width map outperforms the other FCD features in detecting blurred GWB. Furthermore, applying multiple features in FCD detection is ideal.

## Ethics statement

The images were acquired at Ghent University Hospital. All subjects were processed to be anonymous before this study to protect their privacy. The data used in this study were extracted from a retrospective study that was approved by the local Ethics Committee of the Ghent University Hospital. The 10 patients and 31 healthy people involved in our study have provided written consent. All patients suffered from epilepsy due to FCD have been confirmed by clinical examinations.

## Author contributions

XQ designed the work, made the experiments, analyzed the data sets and wrote the manuscript. JY finally revise the manuscript and make approval of the version to be published. DA modified the manuscript after HS and LZ, and checked all the figures and tests. HS and LZ revised the manuscript for several times and gave promising suggestions. YW, TB, and WP designed the topic of the work and provided the framework of the topic.

### Conflict of interest statement

The authors declare that the research was conducted in the absence of any commercial or financial relationships that could be construed as a potential conflict of interest.

## Footnotes

^1^http://www.itksnap.org/pmwiki/pmwiki.php

^2^http://neuro.debian.net/pkgs/fsl-mni152-templates.html

## Abbreviations

FCD: Focal Cortical Dysplasia
MR: Magnetic Resonance
LDPO: Local Directional Probability Optimization
GWB: Gray/White matter Boundary
GM: Gray Matter
WM: White Matter
CSF: Cerebral Spinal Fluid
HMRF–EM: Hidden Markov Random Field–Expectation–Maximization
MRF: Markov Random Field
EM: Expectation–Maximization
ROC: Receiver Operating Characteristic
TPR: True Positive Rate
FPR: False Positive Rate
TP: True Positive
TN: True Negative
FP: False Positive
FN: False Negative
MNI: Montreal Neurological Institute.
